# Factors associated with sleep problems and sleeping pill use in Brazilians

**DOI:** 10.11606/s1518-8787.2022056004088

**Published:** 2022-07-22

**Authors:** Mayonara Fabíola Silva Araújo, Talita Araújo de Souza, Arthur de Almeida Medeiros, Jane Carla de Souza, Isabelle Ribeiro Barbosa

**Affiliations:** I Universidade Federal do Rio Grande do Norte Programa de Pós-graduação em Saúde Coletiva Natal RN Brasil Universidade Federal do Rio Grande do Norte . Programa de Pós-graduação em Saúde Coletiva . Natal , RN , Brasil; II Universidade Federal do Rio Grande do Norte Programa de Pós-graduação em Ciências da Saúde Natal RN Brasil Universidade Federal do Rio Grande do Norte . Programa de Pós-graduação em Ciências da Saúde . Natal , RN , Brasil; III Universidade Federal do Mato Grosso do Sul Instituto Integrado de Saúde Campo Grande MS Brasil Universidade Federal do Mato Grosso do Sul . Instituto Integrado de Saúde . Campo Grande , MS , Brasil; IV Universidade Federal do Rio Grande do Norte Faculdade de Ciências da Saúde do Trairi Santa Cruz RN Brasil Universidade Federal do Rio Grande do Norte . Faculdade de Ciências da Saúde do Trairi . Santa Cruz , RN , Brasil

**Keywords:** Sleep Initiation and Maintenance Disorders, epidemiology, Sleep Aids, Pharmaceutical, therapeutic use, Risk Factors, Health Surveys

## Abstract

**OBJECTIVE:**

To estimate the prevalence of sleep problems and sleeping pill use and associated factors in the Brazilian population.

**METHODS:**

This study was conducted with data from the 2019 Brazilian National Health Survey. Our sample consisted of 94,114 participants and the outcomes analyzed were sleep problems and sleeping pill use. Sociodemographic, lifestyle, and health characteristics were explored in a descriptive and multivariate analysis with Poisson regression, robust variance, and 5% significance.

**RESULTS:**

We found a 35.1% (95%CI: 34.5–35.7) and 8.5% (95%CI: 8.2–8.9) prevalence of sleep problems and sleeping pill use, respectively. Sleep problems were associated with women (PR = 1.41; 95%CI: 1.36–1.46), individuals who self-assess their health as regular/poor/very poor (PR = 1.56; 95%CI: 1.51–1.62), those with chronic diseases (PR = 1.70; 95%CI: 1.64–1.78), those who use alcohol excessively (PR = 1.14; 95%CI: 1.09–1.20), and smokers (PR = 1.16; 95%CI: 1.10–1.22). Sleeping pill use was associated with women (PR = 1.57; 95%CI: 1.43–1.73), divorcees (PR = 1.46; 95%CI: 1.30–1.65), urban denizens (PR = 1.32; 95%CI: 1.21–1.45) those who self-assess their health as regular/poor/very poor (PR = 1.79; 95%CI: 1.64–1.95), those with chronic diseases (PR = 4.07; 95%CI: 3.48–4.77), and smokers (PR = 1.49; IC95%: 1.33–1.67).

**CONCLUSION:**

This study found that the prevalence of sleep problems and sleeping pill use in Brazilians indicates the need for attention and sleep care for this population, especially in women and those with lifestyle and health conditions associated with the analyzed outcomes.

## INTRODUCTION

Sleep is a biological, complex, and active process which is considered essential for life and the maintenance of human health ^1^ . The body performs important functions during sleep, such as restoring physiological systems after wakefulness, conserving and restoring energy metabolism, strengthening the immune system, secreting hormones, consolidating memories, and maintaining neuronal integrity ^1^ .

Thus, poorly slept nights negatively interfere in organ and system function, impairing individuals’ quality of life and general well-being ^2^ , contributing to the emergence of several diseases ^3^ . Moreover, sleep alterations can significantly impact work productivity, increase individuals’ tendency to errors and accidents due to impaired concentration capacity ^4^ , and increase work absenteeism ^2^ .

Besides the absence of sleep disorders, maintaining a good sleep duration and a regular sleep-wake schedule are necessary to ensure sleep functions and benefits. However, these factors vary throughout life and from person to person ^2^ .

Nowadays, lifestyle factors such as work overload, intense task routines, stress, technology use, and exposure to numerous contemporary digital stimuli near bedtime are related to shorter and poorer sleep, excessive daytime sleepiness, and sleeping pill use to attempt to solve these problems ^5^ .

An international survey with 10,132 participants found that 56% of individuals in the United States, 31% in Western Europe, and 23% in Japan showed sleep problems ^6^ . In Colombia, 59.6% of interviewees ^7^ had sleep complaints. In Brazil, the Brazilian Sleep Society found an increase in the percentage of people who reported sleep problems, from 56.7% in 2018 to 60.4% in 2019 ^8^ .

Thus, various populations, ages, and ethnicities suffer from sleep problems, and many people seek medicine use as an intervention strategy. The best known and often used sleeping pill are benzodiazepine hypnotics, sedatives, and anxiolytics, which decrease sleep latency, make individuals fall asleep faster, reduce nocturnal awakening, and increase sleep duration. However, prolonged and undue use of these drugs brings important adverse effects, including dependence and abuse risks ^9^ .

In the United States, the number of sleeping pill prescriptions increased by 293% in a decade, from 5.3 million in 1999 to 20.8 million in 2010 ^10^ . In Brazil, 7.6% of its population used sleeping pills in 2013 ^11^ . Moreover, the prevalence of sleeping pill use in older adults (aged 75 years or older) in a Brazilian city increased from 24.9% in 1997 to 33.9% in 2012 ^9^ .

In this context, considering how important sleep is for people’s health and quality of life, this study aimed to estimate the prevalence of, and factors associated with sleep problems and sleeping pill use in the Brazilian population, based on data from the 2019 National Health Survey ( *Pesquisa Nacional de Saúde* - PNS 2019). In this study, sleep problems refer to difficulty falling asleep (a symptom of insomnia), frequent nocturnal awakening (sleep fragmentation), and sleeping longer than usual (a result of sleep deprivation). The results of this current population-based research can serve as basis for the formulation of strategies and policies in the areas of promotion, surveillance, and health care and welfare of the Brazilian population.

## METHODS

This study was conducted with data from the Brazilian National Health Survey ( *Pesquisa Nacional de Saúde* – PNS 2019), conducted between 2019 and 2020. PNS is a household population survey carried out by the *Instituto Brasileiro de Geografia e Estatística* (IBGE – Brazilian Institute of Geography and Statistics) in partnership with the Ministry of Health, aiming to assess what determines and conditions the health of Brazilians and their needs in a representative database of the Brazilian population.

Data was collected via PNS home interviews and sampling, using the *Amostra Mestra do Sistema Integrado de Pesquisa Domiciliares* (Master Sample of the Integrated System of Household Surveys), which allows greater territorial coverage and uses a three-stage cluster sampling process with a simple random sample. Its first stage consists of Primary Sampling Units (census tracts), the second one integrates the selected households, and the third one includes the residents aged 15 years old or above who were selected in each household to answer the survey.

At the end, 108,457 households were selected, of which 100,541 were occupied and, of these, 94,114 people were chosen as volunteers ^12^ . The final sample of this study consists of the 94,114 interviewed individuals aged 15 years old and above.

This study had two dependent variables: “sleep problems,” assessed by affirmative or negative answered to question “N010 – *In the last two weeks, did you have sleep problems such as difficulty falling asleep, waking up often at night or sleeping more than usual?”* and “sleeping pill use,” also assessed by affirmative and negative answers to question “Q132 – *Did you use any* sleeping pill *in the last two weeks?* ”

Our independent sociodemographic individual variables were gender (male or female), age (15–29, 30–59, 60 years or more), race/skin color (white, Black – Black and Brown –, and Indigenous or Asian), marital status (married, divorced, widowed or single), educational attainment (illiterate/no schooling, complete or incomplete primary education, complete or incomplete secondary education, and complete or incomplete higher education), *per capita* household income (up to one minimum wage, one to three minimum wages, and above three minimum wages), area of residence (urban or rural), household size (one, two to three, or four or more residents), and Family Health Strategy - FHS – coverage (yes, no or does not know).

**Figure 1:**
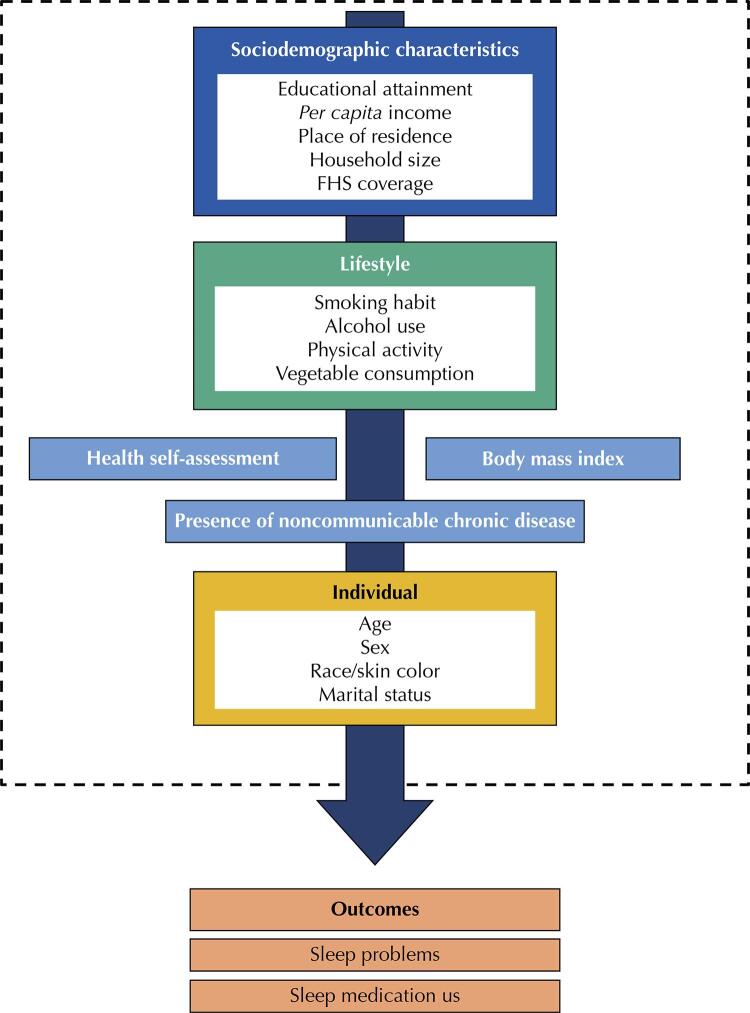
Study framework.

The following lifestyle and health variables were considered: health self-assessment (very good/good; regular/poor/very poor); diagnosis of chronic, physical or mental disease, chronic health condition or long-term disease (yes, no); use of tobacco and its derivatives (smoker – currently smokes some tobacco product; former smoker – smoked some tobacco product in the past; never smoked); alcohol use (excessive use – five or more daily 50-mL doses (considered the standard) in at least a single occasion in the last 30 days; moderate use – habitual use regardless of the dose consumed in the last 30 days, but lower than excessive use; and teetotalism); vegetable consumption frequency (less than five days a week, five or more days a week); body mass index (malnutrition, eutrophy, overweight, and obesity); and weekly exercise or sports practice (in minutes). The conceptual model of dependent and independent variables are shown in Figure 1 .

FHS: Family Health Strategy.

Since this is a study with complex sampling, sample weight was incorporated into its design. The calculation was carried out of the prevalence of outcomes in relation to individual variables, with presentation of the respective 95% confidence intervals (95%CI).Then, a bivariate Poisson regression was performed to estimate crude prevalence ratios (PR) and 95% confidence intervals (95%CI).

Variables showing p ≤ 0.20 in the bivariate analysis were included in the multivariate Poisson regression model to estimate adjusted prevalence (PR) ratios. A hierarchical model was adopted and variables were inserted into the multivariate model as their p-values increased. Only statistically significant variables (p < 0.05) were kept in the final model. All analyses were made in the Stata software version 13 (Stata Corp., College Station, United States).

The 2019 Brazilian National Health Survey was approved by the National Committee of Ethics in Research (CONEP) at the Ministry of Health National Council of Health (CNS) under opinion no. 3.529.376, of August 23, 2019. The results of that research are in public domain and available at the *Instituto Brasileiro de Geografia e Estatística* website.

## RESULTS

**Figure 2:**
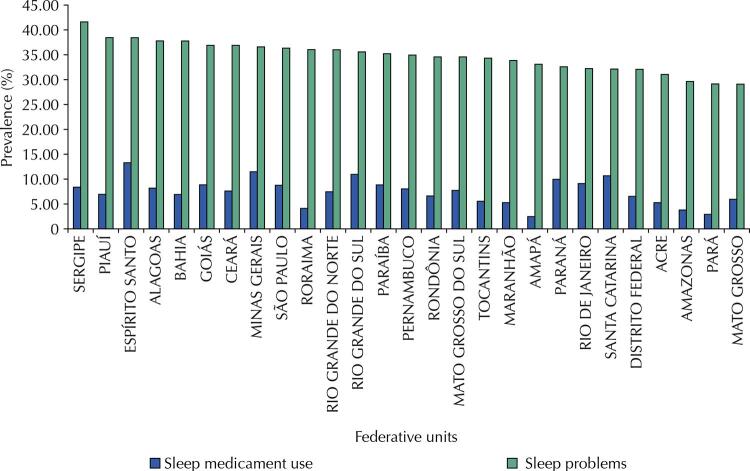
Prevalence of sleep problems and sleep medicament use (n = 94,114) in Brazilian federative units. National Health Survey, 2019.

The Brazilian population shows a 35.1% (95%CI 34.5–35.7) sleep problem prevalence. Sergipe (41.60%), Piauí (38.45%), and Espírito Santo (38.43%) were the Brazilian federative units with the largest sleep problem prevalence ratios ( Figure 2 ).

We found an 8.5% (95%CI 8.2–8.9) prevalence of sleeping pill use. Espírito Santo (13.29%), Minas Gerais (11.48%), and Rio Grande do Sul (10.96%) were the Brazilian federative units with the largest sleeping pill use prevalence ratios ( Figure 2 ).

Our descriptive analysis showed a higher prevalence of sleep problems among women (41.84%), Indigenous people (39.95%), widowers (46.19%), people with no education (43.52%), those who live alone (41%), urban citizens (35.92%), those not registered in the FHS (35.62%), those who assess their health as regular/poor/very poor (51.38%), those with chronic diseases (47.51%), teetotalers (35.98%), former smokers (40.31%), those who consume vegetables less than five times a week (35.48%), those aged 60 years or older (42.05%), those with a *per capita* income above three minimum wages (36.35%), and people with obesity (39.04%). In addition, the mean (in minutes) of physical activity practice was 48.69 (± 1.20) ( Table 1 ).

**Table 1:** Prevalence of sleep problems, crude and adjusted prevalence ratios between outcome and Brazilians’ sociodemographic variables, lifestyle, and health conditions. National Health Survey, 2019.

Variables	n	Descriptive		Bivariate analysis			Multivariate analysis		
		Prevalence	95%CI	_crude_ PR	95%CI	p	_adjusted_ PR	95%CI	p
Gender									
Male	44,752	27.50	26.65–28.36	1			1		
Female	49,362	41.84	41.03–42.64	1.52	1.47–1.57	< 0.005	1.41	1.36–1.46	< 0.005
Race/skin color									
White	34,320	36.15	35.19–37.12	1			1		
Black	58,390	34.34	33.56–35.11	0.94	0.91–0.98	0.020	0.96	0.92–0.99	0.019
Asian	692	29.42	23.05–36.70	0.81	0.63–1.03	0.100	0.79	0.62–1.01	0.071
Indigenous	702	39.95	32.80–47.56	1.10	0.91–1.33	0.310	1.04	0.88–1.23	0.592
Marital status									
Single	42,325	32.04	31.12–32.95	1			1		
Married	36,354	35.46	34.56–36.36	1.10	1.06–1.14	< 0.005	0.99	0.95–1.02	0.66
Divorced	7,713	42.81	40.79–44.84	1.33	1.26–1.40	< 0.005	1.05	1.00–1.11	0.027
Widower	7,722	46.19	44.31–48.07	1.44	1.37–1.51	< 0.005	0.96	0.91–1.01	0.165
Educational attainment									
Higher education	18,287	36.08	34.71–37.46	1			1		
Secondary education	31,128	31.38	30.38–32.39	0.86	0.83–0.91	< 0.005	0.89	0.85–0.93	< 0.005
Primary education	36,829	36.94	35.97–37.92	1.02	0.98–1.06	0.270	0.88	0.84–0.92	< 0.005
Illiterate/no formal education	7,870	43.52	41.44–45.60	1.20	1.13–1.28	< 0.005	0.94	0.88–1.01	0.120
Household size									
1 resident	14,760	41.00	39.72–42.28	1					
2–3 residents	49,219	36.06	35.31–36.81	0.87	0.84–0.91	< 0.005			
> 4 residents	30,135	32.97	31.96–33.99	0.80	0.77–0.83	< 0.005			
Area of residence									
Rural	21,405	30.03	28.95–31.12	1			1		
Urban	72,709	35.92	35.21–36.63	1.19	1.15–1.24	< 0.005	1.16	1.11–1.20	< 0.005
FHS Coverage									
Yes	59,358	35.25	34.52–35.97	1					
No	23,424	35.62	34.32–36.93	1.01	0.97–1.04	0.589			
Does not know	11,332	33.03	31.39–34.71	0.93	0.88–0.98	0.015			
Health self-assessment									
Very good/good	60,055	27.65	26.92–28.38	1			1		
Regular/poor/very poor	34,059	51.38	50.34–52.40	1.85	1.80–1.91	< 0.005	1.56	1.51–1.62	< 0.005
Has NCD									
No	37,093	23.15	22.33–23.98	1			1		
Yes	44,125	47.51	46.64–48.37	2.05	1.97–2.13	< 0.005	1.70	1.64–1.78	< 0.005
Alcohol use									
Teetotalism	55,430	35.98	35.24–36.72	1			1		
Moderate use	20,384	33.92	32.69–35.15	0.94	0.90–0.98	0.003	1.02	0.98–1.07	0.196
Excessive use	15,032	33.70	32.17–35.26	0.93	0.89–0.97	0.004	1.14	1.09–1.20	< 0.005
Smoking habit									
Never smoked	55,236	32.17	31.44–32.90	1			1		
Former smoker	24,224	40.31	39.08–41.54	1.25	1.20–1.29	< 0.005	1.11	1.07–1.15	< 0.005
Smoker	11,386	38.80	37.15–40.47	1.20	1.25–1.26	< 0.005	1.16	1.10–1.22	< 0.005
Weekly vegetable consumption									
≥ 5	46,754	35.48	34.64–36.33	1			1		
< 5	44,092	34.64	33.74–35.53	0.96	0.93–0.99	0.029	1.06	1.02–1.09	< 0.005
Age									
15–29 years old	18,648	27.44	26.18–28.72	1					
30–59 years old	52,322	36.22	35.45–36.99	1.32	1.25–1.38	< 0.005			
≥ 60 years old	23,144	42.05	40.93–43.17	1.53	1.45–1.61	< 0.005			
*Per capita* income									
Up to 1 minimum wage	10,964	35.18	34.38–35.98	0.96	0.90–1.00	0.186			
1 to 3 minimum wages	31,109	34.60	33.60–35.61	0.95	0.92–1.01	0.062			
Above 3 minimum wages	52,017	36.35	34.70–38.03	1					
BMI									
Malnutrition	2,197	34.47	30.62–38.52	1.05	0.93–1.18	0.366			
Eutrophic	36,356	32.68	31.74–33.62	1					
Overweight	32,972	35.46	34.51–36.42	1.08	1.04–1.12	< 0.005			
Obesity	18,106	39.04	37.82–40.26	1.19	1.14–1.24	< 0.005			
Weekly physical activity (minutes)	94,114	48.69 ± 1.20	46.33–51.04	1.09	1.03–1.15	0.003			

_crude_ PR: crude prevalence ratio; _adjusted_ PR: adjusted prevalence ratio; 95%CI: 95% confidence interval; FHS: family health strategy; NCD: chronic non-communicable diseases; BMI: body mass index.

The analysis of the association between sleep problems and sociodemographic, lifestyle and health characteristics of Brazilians in the bivariate analysis showed that all variables surveyed had p < 0.20 and were included in the multivariate regression model ( Table 1 ).

In the final model of our multivariate analysis, the highest prevalence of sleep problems was associated with women (PR = 1.41), divorcees (PR = 1.05), urban denizens (PR = 1.16), those who assess their health as regular/poor/very poor (PR = 1.56), those with chronic diseases (PR = 1.70), those who use alcohol excessively (PR = 1.14), smokers (PR = 1.16) or former smokers (PR = 1.11), and those who consume vegetables less than five times a week (PR = 1.06). Moreover, black ethnicity (PR = 0.96) and complete secondary (PR = 0.89) or primary education (PR = 0.88) were associated with a lower prevalence of sleep problems ( Table 1 ).

Our descriptive analysis showed that the prevalence of sleeping pill use was higher among women (11.51%), white individuals (10.37%), widowers (18.32%), people with no education (13,44%), those living alone (12.68%), urban denizens (8.9%), those registered in the FHS (8.68%), those who assess their health as regular/poor/very poor (15.72%), those with chronic diseases (15.2%), teetotalers (10.39%), smokers (10.48%), those who consume vegetables five times or more per week (9.13%), those aged 60 years or older (15.27%), people with obesity (10.54%), and those with a *per capita* income above three minimum wages (10.38%). We found an average 53.86 minutes (± 2.47) of physical activity in our sample ( Table 2 ).

**Table 2:** Prevalence of sleeping pill use, crude and adjusted prevalence ratios between outcome and Brazilians’ sociodemographic variables, lifestyle, and health conditions. National Health Survey, 2019.

Variables	n	Descriptive		Bivariate analysis			Multivariate Analysis		
		Prevalence	95%CI	_crude_ PR	95%CI	p	_adjusted_ PR	95%CI	p
Gender									
Male	44,752	5.21	4.84–5.59	1			1		
Female	49,362	11.51	11.02–12.03	2.21	2.03–2.40	< 0.005	1.57	1.43–1.73	< 0.005
Race/skin color									
White	34,320	10.37	9.79–10.98	1			1		
Black	58,390	7.18	6.82–7.56	0.69	0.64–0.74	< 0.005	0.72	0.67–0.78	< 0.005
Asian	692	6.10	3.37–10.79	0.58	0.31–1.08	0.088	0.55	0.31–0.98	0.044
Indigenous	702	8.68	5.55–13.33	0.83	0.54–1.29	0.423	0.78	0.53–1.15	0.222
Marital status									
Single	42,325	5.61	5.21–6.04	1			1		
Married	36,354	9.06	8.56–9.22	1.61	1.47–1.77	< 0.005	1.03	0.93–1.13	0.536
Divorced	7,713	15.77	14.31–17.35	2.81	2.48–3.17	< 0.005	1.46	1.30–1.65	< 0.005
Widower	7,722	18.32	16.87–19.87	3.26	2.92–3.64	< 0.005	1.19	1.06–1.34	< 0.005
Educational attainment									
Higher education	18,287	8.37	7.59–9.22	1			1		
Secondary education	31,128	6.04	5.57–6.55	0.72	0.63–0.81	< 0.005	0.80	0.71–0.91	0.001
Primary education	36,829	10.36	9.81–10.93	1.23	1.11–1.37	< 0.005	0.91	0.81–1.02	0.112
Illiterate/no formal education	7,870	13.44	12.02–15.01	1.60	1.39–1.85	< 0.005	0.95	0.81–1.12	0.594
Household size									
1 resident	14,760	12.68	11.88–13.54	1					
2–3 residents	49,219	9.68	9.23–10.14	0.76	0.70–0.82	< 0.005			
> 4 residents	30,135	6.51	6.02–7.04	0.51	0.46–0.57	< 0.005			
Area of residence									
Rural	21,405	6.37	5.84–6.93	1			1		
Urban	72,709	8.90	8.54–9.28	1.39	1.27–1.52	< 0.005	1.32	1.21–1.45	< 0.005
FHS coverage									
Yes	59,358	8.68	8.29–9.08	1					
No	23,424	8.46	7.77–9.20	0.97	0.89–1.06	0.572			
Does not know	11,332	8.03	7.11–9.07	0.92	0.80–1.06	0.269			
Health self-assessment									
Very good/good	60,055	5.26	4.93–5.62	1			1		
Regular/poor/very poor	34,059	15.72	15.02–16.45	2.98	2.75–3.23	< 0.005	1.79	1.64–1.95	< 0.005
Has NCD									
No	37,093	2.41	2.10–2.77	1			1		
Yes	44,125	15.20	14.59–15.82	6.29	5.42–7.31	< 0.005	4.07	3.48–4.77	< 0.005
Alcohol use									
Teetotalism	55,430	10.39	9.95–10.85	1			1		
Moderate use	20,384	6.65	6.03–7.33	0.63	0.57–0.71	< 0.005	0.75	0.67–0.84	< 0.005
Excessive use	15,032	4.79	4.20–5.45	0.46	0.40–0.52	< 0.005	0.68	0.59–0.78	< 0.005
Smoking habit									
Never smoked	55,236	7.41	7.04–7.79	1			1		
Former smoker	24,224	10.35	9.69–11.06	1.39	1.28–1.52	< 0.005	1.15	1.05–1.25	0.002
Smoker	11,386	10.48	9.48–11.56	1.41	1.26–1.58	< 0.005	1.49	1.33–1.67	< 0.005
Weekly vegetable consumption									
≥ 5 times	46,754	9.31	8.84–9.81	1					
< 5 times	44,092	7.64	7.21–8.09	0.81	0.75–0.88	< 0.005			
Age									
15–29 years old	18,648	3.03	2.58–3.55	1			1		
30–59 years old	52,322	8.70	8.26–9.17	2.87	2.43–3.39	< 0.005	1.50	1.26–1.79	< 0.005
≥ 60 years old	23,144	15.27	14.47–16.10	5.04	4.27–5.96	< 0.005	1.50	1.24–1.81	< 0.005
*Per capita* income									
Up to 1 minimum wage	10,964	7.94	7.52–8.38	0.76	0.68–0.84	< 0.005			
1 to 3 minimum wages	31,109	8.86	8.29–9.46	0.85	0.76–0.95	0.006			
Above 3 minimum wages	52,017	10.38	9.41–11.44	1					
BMI									
Malnutrition	2,197	7.61	5.91–9.75	1.03	0.78–1.35	0.823			
Eutrophic	36,356	7.38	6.91–7.87	1					
Overweight	32,972	9.05	8.50–9.64	1.22	1.12–1.34	0.006			
Obesity	18,106	10.54	9.82–11.31	1.42	1.29–1.58	0.006			
Weekly physical activity (minutes)	94,114	53.86 ± 2.47	49.00–58.72	1.18	1.08–1.30	< 0.005			

_crude_ PR: crude prevalence ratio; _adjusted_ PR: adjusted prevalence ratio; 95%CI: 95% confidence interval; FHS: family health strategy; NCD: chronic non-communicable diseases; BMI: body mass index.

The bivariate analysis of the association between sleep problems and Brazilians’ sociodemographic, lifestyle, and health characteristics showed that “FHS coverage” was the only variable which failed to show a p < 0.20. We included all other variables in the multivariate regression model.

Our final multivariate analysis model associated the higher prevalence of with women (PR = 1.57), divorcees (PR = 1.46), widowers (PR = 1.19), urban denizens (PR = 1.32), those who assess their health as regular/poor/very poor (PR = 1.79), those with chronic diseases (PR = 4.07), former smokers (PR = 1.15), smokers (PR = 1.49), and those aged 30 to 59 years old (PR = 1.50) and 60 years or older (PR = 1.50). Black (PR = 0.77) and Asian individuals (PR = 0.55), those with complete secondary education (PR = 0.80), those moderately using alcohol (PR = 0.75), and those who excessively use alcohol (PR = 0.68) were associated with a lower prevalence of sleeping pill use ( Table 2 ).

## DISCUSSION

The prevalence of Brazilians aged 15 years old and older who reported sleep problems, such as difficulty falling asleep, frequent nocturnal awakening or sleeping more than usual, and used sleeping pill in the two weeks prior to research, were 35.1% and 8.5%, respectively. These prevalence ratios are higher than the 2013 PNS ones, in which 28.9% of interviewees reported sleep problems ^13^ and 7.6% used sleeping pill ^11^ .

Corroborating Brazilian studies, both outcomes analyzed in this study were more common in women ^14^ , divorcees ^15 , 16^ , urban denizens ^17^ , those who negatively self-assess their health (regular/poor/very poor) ^16 , 18^ , those with chronic diseases ^16 , 18^ , smokers, and former smokers ^11^ .

The literature has widely discussed the factors associated with sleep-related differences between genders but found no consensus ^19^ . Some authors relate women’s more fragmented sleep to hormonal and physiological variations ^19^ or to social issues to which they are exposed, such as greater social demands and overload at work and at home ^20^ .

In general, women report a greater need for sleep and more subjective complaints of nonrestorative sleep than men ^19^ , which may have contributed to more prevalent medicine use. Moreover, women also show better adherence to treatment and self-care, which may be related to greater adherence to medicine use ^20^ .

Corroborating other Brazilian studies, we found a higher prevalence of sleep problems ^15^ and sleeping pill use ^16^ in divorcees. Lamela ^21^ (2009) conducted a literature review which considered divorce one of the most impacting stressors in adulthood since those who experience it suffer changes in many areas of their lives in a short period of time, becoming more prone or vulnerable to psychological symptoms. This may explain their higher prevalence of difficulty sleeping, frequent nocturnal awakening, unusually long sleep, and sleeping pill use.

We also found that living in urban areas is a factor impacting Brazilians’ sleep. The intensity of artificial lights in urban areas, especially at nighttime, strongly influences sleep duration and sleep-wake schedules ^22^ . Artificial light causes changes in the body, such as melatonin suppression - a hormone which signals nighttime and induces sleep in humans - and increased nervous system activity, resulting in greater alertness and subsequent difficulty falling asleep ^17^ .

The higher prevalence ratios of both outcomes were also associated with people who assess their health as regular/poor/very poor. Health self-assessment and self-reported sleep problems are closely related, i.e., satisfaction with the way one sleeps is one of the main factors in health self-assessment ^23^ . Studies with young Brazilians ^23^ found that self-reporting good sleep increased the probability of better health self-assessments.

On the other hand, a study with a representative sample, conducted with Brazilians aged 50 years or older, found an association between participants self-assessing their health as poor with greater sleeping pill use ^16^ . The same study shows that the presence of chronic diseases was associated with a higher prevalence of medicine use ^16^ , as did we.

In general, chronic diseases generate inflammations in the body and can cause pain, emotional changes, and other consequences due to unrestored physiological systems after wakefulness events ^24^ . Diseases such as hypertension, osteoporosis, arthritis/arthrosis, low back pain, depression, and obesity are associated with sleep problems ^24^ . Moreover, people who showed five or more concomitant health problems had a 4.19% higher prevalence of sleep problems than those with no or fewer comorbidities ^18^ .

Individuals who reported smoking or having smoked also had a higher prevalence in the studied outcomes. Nicotine, the main substance in cigarettes, is a stimulant associated with changes in sleep architecture, fragmenting it and decreasing its efficiency ^25^ .

Moreover, a higher prevalence of sleep problems was associated with excessive alcohol use, which has harmful consequences for individuals’ overall health. Excessive alcohol consumption interferes with the physiology of sleep, especially in the second half of the night, facilitating awakenings, which fragments sleep ^26^ , reflecting poor sleep quality.

A Brazilian population-based cross-sectional study which used 2013 PNS ^11^ data found a lower prevalence of sleeping pill use associated with moderate or excessive use of alcohol, which may be due to its hypnotic effect, depressing the central nervous system, reducing sleep latency, and facilitating sleep ^26^ .

Inadequate eating habits and sleep disorders, bedtime, and poor sleep quality and duration ^27^ are another important discussion factor. One example is the association between increased fruit and vegetable consumption with improved sleep quality and duration ^27^ , which may relate to the association we found, i.e., a higher prevalence of sleep problems in individuals who reported consuming vegetables less than five times a week.

We observed an association with individuals aged over 30 years old and a higher prevalence of sleeping pill use. Considering that the age group composed by 30 to 59 year-olds has the highest work and social activity ^2^ , they may use sleeping pill in an attempt to improve sleep quality, reduce insomnia and anxiety, and feel relaxed ^11^ .

Moreover, a Brazilian population-based study conducted with 2013 PNS data found that 21% of respondents reported depressive symptoms and that sleeping pill use was associated with people with these symptoms, which may justify the high prevalence in its use ^11^ .

The literature has observed that people aged 60 years old and above undergo physiological changes to sleep quality, quantity, and architecture as age advances, resulting in a more superficial, shorter, worse, and fragmented sleep ^28^ .

Pharmacological therapy is the most used among older adults ^20^ . In general, the use of medicine minimizes sleep alterations such as trouble falling asleep and waking throughout the night ^16^ . However, much is discussed about the long-term use of these medicines, their side effects, and the large quantity of drugs consumed by older adults ^20^ .

In this study, Black individuals were associated with a lower prevalence of sleep problems and sleeping pill use. This result corroborates an epidemiological study conducted in the United States in which white adults report taking more sleeping pill than Black ones ^29^ .

However, the literature indicates that Black individuals are prone to worse, shorter, and more fragmented sleep, showing a higher risk of sleep breathing disorders than white individuals ^30^ . Despite these results, little is known about the mechanism behind ethnic sleep differences ^30^ .

Lower prevalence of sleep problems was also associated with having attended primary or high school and the lower prevalence of sleeping pill use was associated with having attended high school. Thus, the literature believes that people with higher educational attainment have the necessary knowledge on beneficial health behaviors and better habits close to bedtime, thus reducing sleep-related complaints and resulting in a lower need for sleeping pill ^16^ .

In view of the above, this study shows important potentialities since it is a representative national population-based study on sleep problems and sleeping pill in Brazil, including more than 90,000 participants aged 15 years old and above and evaluating their sociodemographic, lifestyle, and health characteristics.

However, a limitation of this study was the impossibility of identifying in greater detail from what sleep problems Brazilians suffer and what medicine they use, considering that the answers to the PNS are self-reported and our analysis ignored the evaluation of these specifications. It is important to remember that the interviewers were trained to apply the questionnaires but since this information is self-reported and questions refer to behaviors from previous weeks, it is possible that answers suffered from memory biases and differences in interviewees’ understanding.

Finally, the prevalence of sleep problems and sleeping pill use was high in the Brazilian population, an important datum for public health which shows the need for attention and care for sleep in this population. Our multivariate analysis showed the associations between sleep problems and sleeping pill use and various sociodemographic, health, and lifestyle factors. Thus, the results of this study add updated evidence of the relation between sleep and social factors and suggest more specific, further research to characterize Brazilians’ sleep problems, types, and frequency and their use of sleeping pill.
