# Supplementary material for: Factors associated with sleep problems and sleeping pill use in Brazilians
Source: Rev Saude Publica. 2022 Jul 22;56:68. doi: 10.11606/s1518-8787.2022056004088 (PMC9337849; doi:10.11606/s1518-8787.2022056004088)
Supplement: Translation

# Translation: Fatores associados aos problemas de sono e ao uso de medicação para dormir em brasileiros

**Authors:** Mayonara Fabíola Silva Araújo, Talita Araújo de Souza, Arthur de Almeida Medeiros, Jane Carla de Souza, Isabelle Ribeiro Barbosa

**Affiliations:** I Brasil Universidade Federal do Rio Grande do Norte. Programa de Pós-graduação em Saúde Coletiva. Natal, RN, Brasil; II Brasil Universidade Federal do Rio Grande do Norte. Programa de Pós-graduação em Ciências da Saúde. Natal, RN, Brasil; III Brasil Universidade Federal do Mato Grosso do Sul. Instituto Integrado de Saúde. Campo Grande, MS, Brasil; IV Brasil Universidade Federal do Rio Grande do Norte. Faculdade de Ciências da Saúde do Trairi. Santa Cruz, RN, Brasil

**Keywords:** Distúrbios do Início e da Manutenção do Sono, epidemiologia, Medicamentos Indutores do Sono, uso terapêutico, Fatores de Risco, Inquéritos Epidemiológicos

## Abstract

**OBJETIVO:**

Estimar a prevalência e fatores associados a problemas de sono e uso de medicamentos para dormir na população brasileira.

**MÉTODOS:**

Estudo executado com os dados da Pesquisa Nacional de Saúde realizada no Brasil, nos anos de 2019 e 2020. A amostra foi composta por 94.114 participantes e os desfechos analisados foram problemas de sono e uso de medicamentos para dormir. Aspectos sociodemográficos, de estilo de vida e condições de saúde foram explorados em uma análise descritiva e multivariada, utilizando a regressão de Poisson com variância robusta, considerando nível de significância de 5%.

**RESULTADOS:**

As prevalências de problemas de sono e uso de medicamentos indutores do sono foram de 35,1% (IC95% 34,5–35,7) e 8,5% (IC95% 8,2–8,9), respectivamente. Os problemas de sono foram associados ao sexo feminino (RP = 1,41; IC95% 1,36–1,46), aos indivíduos que autoavaliam a saúde como regular/ruim/muito ruim (RP = 1,56; IC95% 1,51–1,62), aos que possuem alguma doença crônica (RP = 1,70; IC95% 1,64–1,78), aos que fazem uso excessivo de álcool (RP = 1,14; IC95% 1,09–1,20) e aos fumantes (RP = 1,16; IC95% 1,10–1,22). O uso de medicamentos para dormir foi associado ao sexo feminino (RP = 1,57; IC95% 1,43–1,73), a indivíduos divorciados (RP = 1,46; IC95% 1,30–1,65), aos que vivem no meio urbano (RP = 1,32; IC95% 1,21–1,45), que autoavaliam sua saúde como regular/ruim/muito ruim (RP = 1,79; IC95% 1,64–1,95), com diagnóstico de doença crônica (RP = 4,07; IC95% 3,48–4,77) e aos fumantes (RP = 1,49; IC95% 1,33–1,67).

**CONCLUSÃO:**

As prevalências de problemas de sono e uso de medicamentos para dormir na população brasileira observadas neste estudo indicam a necessidade de atenção e cuidado com o sono dessa população, principalmente nas mulheres e aqueles que apresentam estilo de vida e condições de saúde que se associaram aos desfechos.

## INTRODUÇÃO

O sono é um processo biológico, complexo, ativo e considerado essencial para a vida e a manutenção da saúde humana ^1^ . É durante o sono que o organismo realiza funções importantes, como o restabelecimento dos sistemas fisiológicos após os eventos de vigília, conservação e restauração do metabolismo energético, fortalecimento do sistema imunológico, secreção de alguns hormônios, consolidação de memórias e manutenção da integridade neuronal ^1^ .

Portanto, noites de sono mal dormidas interferem negativamente no funcionamento de órgãos e sistemas, trazendo comprometimento da qualidade de vida e bem-estar geral dos indivíduos ^2^ , contribuindo para o surgimento de diversas doenças ^3^ . Além disso, as alterações do sono podem impactar de forma significativa na produtividade do trabalho, aumentar a tendência a erros e acidentes devido ao comprometimento da capacidade de concentração ^4^ e aumentar o absenteísmo no trabalho ^2^ .

Para garantir que as funções e benefícios do sono sejam alcançados, é necessário manter uma duração de sono adequada e de boa qualidade, horários regulares para dormir e acordar e ausência de distúrbios do sono. No entanto, deve-se considerar que todos esses fatores variam ao longo da vida e de pessoa para pessoa ^2^ .

Com a contemporaneidade, fatores relacionados ao estilo de vida como sobrecarga de trabalho, rotina intensa de afazeres, estresse, uso de tecnologias e a exposição aos inúmeros estímulos digitais que a modernidade traz consigo, próximo ao horário de dormir, estão relacionados à menor duração e má qualidade de sono, sonolência diurna excessiva e ao uso de medicações para o sono como tentativa de resolução desses problemas ^5^ .

Pesquisa internacional realizada com 10.132 indivíduos observou uma prevalência de problemas de sono de 56% nos Estados Unidos, 31% na Europa Ocidental e 23% no Japão ^6^ . Na Colômbia, a prevalência de queixas de sono foi de 59,6% dos entrevistados ^7^ . No Brasil, a Sociedade Brasileira do Sono constatou aumento no percentual de pessoas que relataram ter problemas de sono, de 56,7% em 2018 para 60,4% em 2019 ^8^ .

Portanto, os problemas de sono estão presentes nas mais variadas populações, idades e etnias, e muitas pessoas buscam o uso de medicamentos como estratégia de intervenção. As medicações para dormir mais conhecidas e frequentemente utilizadas são os hipnóticos, os sedativos e os ansiolíticos da classe dos benzodiazepínicos. Essas medicações diminuem a latência do sono, fazendo o indivíduo adormecer mais rápido, reduzem os despertares a noite e promovem o aumento do tempo de sono. Porém, o uso prolongado e indevido desses medicamentos traz efeitos adversos importantes, incluindo risco de dependência e abuso ^9^ .

Nos Estados Unidos, o número de prescrições de medicamentos para dormir teve um aumento de 293% em uma década, saindo de 5,3 milhões em 1999 para 20,8 milhões em 2010 ^10^ . No Brasil, a prevalência de uso de pílulas para dormir em 2013 foi de 7,6% da população ^11^ . Além disso, a prevalência de uso de medicamentos para dormir em idosos (de 75 anos ou mais) de uma cidade brasileira aumentou de 24,9% em 1997 para 33,9% em 2012 ^9^ .

Nesse contexto, considerando a grande importância do sono para saúde e a qualidade de vida das pessoas, o objetivo deste estudo foi estimar a prevalência e fatores associados a problemas de sono e uso de medicamentos indutores do sono na população brasileira, a partir dos dados da Pesquisa Nacional de Saúde de 2019 (PNS 2019). Neste estudo, problemas de sono referem-se a dificuldade para adormecer (sintoma de insônia), acordar frequentemente à noite (fragmentação do sono) e dormir mais do que o de costume (resultado de privação de sono). Os resultados desta pesquisa de base populacional atual podem servir de embasamento na formulação de estratégias e políticas nas áreas de promoção, vigilância e assistência à saúde e bem-estar da população brasileira.

## MÉTODOS

Este estudo foi conduzido com os dados da Pesquisa Nacional de Saúde – 2019 (PNS), realizada entre 2019 e 2020, que é um inquérito populacional domiciliar conduzido pelo Instituto Brasileiro de Geografia e Estatística (IBGE), em parceria com o Ministério da Saúde e com objetivo de conhecer os determinantes, condicionantes e necessidades de saúde da população brasileira, compondo uma base de dados representativa da população brasileira.

A coleta de dados ocorreu mediante entrevistas em domicílio e plano amostral da PNS, utilizando a Amostra Mestra do Sistema Integrado de Pesquisa Domiciliares, que permite maior abrangência territorial e utiliza processo de amostragem por conglomerados em três estágios, com amostra aleatória simples. O primeiro estágio é composto pelas Unidades Primárias de Amostragem (setores censitários), o segundo estágio integra os domicílios selecionados e o terceiro estágio inclui os moradores com 15 anos ou mais, os quais foram selecionados em cada domicílio para responder à pesquisa.

Ao final, foram selecionados 108.457 domicílios, dos quais 100.541 domicílios estavam ocupados e, desses, 94.114 pessoas optaram por participar voluntariamente da pesquisa ^12^ . A amostra final do presente estudo foram os 94.114 indivíduos entrevistados, com idade a partir de 15 anos.

Este estudo teve duas variáveis dependentes: “problemas de sono”, respondendo, de forma afirmativa ou negativa, à questão “N010 – *Nas duas últimas semanas, o(a) Sr(a) teve problemas no sono, como dificuldade para adormecer, acordar frequentemente à noite ou dormir mais do que de costume?”* ; e “uso de medicamentos indutores do sono”, que também foi respondida de forma afirmativa ou negativa a questão “Q132 – *Nas últimas duas semanas, o(a) Sr(a) fez uso de algum medicamento para dormir?* ”

Entre as variáveis independentes individuais sociodemográficas, foram considerados o sexo (masculino ou feminino), a idade (15–29 anos, 30–59 anos, 60 anos ou mais), a raça/cor da pele (brancos, negros – pretos e pardos, indígenas ou asiáticos), estado civil (casado, divorciado, viúvo, solteiro), escolaridade (sem escolaridade, nível fundamental completo ou incompleto, nível médio completo ou incompleto, nível superior completo ou incompleto), renda domiciliar *per capita* (até um salário mínimo, de um a três salários mínimos, acima de três salários), área de residência (urbano ou rural), aglomeração domiciliar (um morador; 2–3 moradores; > 4 moradores) e se possui cobertura da Estratégia Saúde da Família – ESF (sim, não ou não sabe).

Em relação ao estilo de vida e condições de saúde foram consideradas as seguintes variáveis: autoavaliação do estado de saúde (muito bom/bom; regular/ruim/muito ruim); se possui diagnóstico de doença crônica, física ou mental, condição crônica de saúde ou doença de longa duração (sim, não); uso de tabaco e seus derivados (fumante – atualmente fuma algum produto do tabaco; ex-fumante – no passado fumou algum produto do tabaco; nunca fumou); uso de bebida alcoólica (uso excessivo – cinco ou mais doses diárias em pelo menos uma única ocasião nos últimos 30 dias, considerando a dose padrão de 50 mL; uso moderado – uso habitual independentemente da dose consumida nos últimos 30 dias, porém inferior ao uso excessivo; e não bebe); frequência do consumo semanal de verduras (menos de cinco dias por semana, cinco ou mais dias por semana); índice de massa corporal (desnutrição, eutrofia, sobrepeso e obesidade) e tempo semanal de prática de exercício físico ou esporte (em minutos). O modelo conceitual das variáveis dependentes e independentes está apresentado na Figura 1 .

**Figura 1:** *Framework* do estudo.

ESF: Estratégia Saúde da Família.

Por se tratar de um estudo com amostragem complexa, para as análises foi utilizado o peso da amostra e incorporado o efeito do desenho da amostra. Procedeu-se o cálculo da prevalência dos desfechos em relação às variáveis individuais, com apresentação dos respectivos intervalos de confiança de 95% (IC95%). Em seguida, foi conduzida análise de regressão de Poisson bivariada para estimar a razão de prevalências (RP) bruta e o IC95%.

As variáveis que apresentaram p ≤ 0,20 na análise bivariada foram incluídas no modelo de regressão de Poisson multivariada, a fim de estimar a razão de prevalências (RP) ajustada. Adotou-se o modelo hierárquico e a entrada das variáveis no modelo multivariado foi feita de acordo com a ordem crescente do valor de p. Permaneceram no modelo final apenas as variáveis que apresentaram significância estatística (p < 0,05). Todas as análises foram realizadas utilizando-se o software Stata versão 13 (Stata Corp., College Station, United States).

O projeto da Pesquisa Nacional de Saúde 2019 foi aprovado pela Comissão Nacional de Ética em Pesquisa (CONEP) do Conselho Nacional de Saúde (CNS) do Ministério da Saúde, sob o Parecer nº 3.529.376, de 23 de agosto de 2019. Os resultados da pesquisa são de domínio público e estão disponíveis no site do IBGE.

## RESULTADOS

A prevalência de problemas de sono na população brasileira foi de 35,1% (IC95% 34,5–35,7). As unidades federativas com maiores prevalências de problemas de sono foram os estados de Sergipe (41,60%), Piauí (38,45%) e Espírito Santo (38,43%) ( Figura 2 ).

**Figura 2:** Prevalência de problemas de sono e do uso de medicamentos indutores do sono (n = 94.114) nas unidades federativas do Brasil. Pesquisa Nacional de Saúde, 2019.

A prevalência do uso de medicamentos indutores do sono foi de 8,5% (IC95% 8,2–8,9). As unidades federativas com maior prevalência de uso de medicamentos para dormir foram Espírito Santo (13,29%), Minas Gerais (11,48%) e Rio Grande do Sul (10,96%) ( Figura 2 ).

A análise descritiva demonstrou que houve uma maior prevalência de problemas do sono entre as mulheres (41,84%), os indígenas (39,95%), viúvos (46,19%), pessoas sem instrução (43,52%), os que moram sozinhos (41%), vivem no meio urbano (35,92%), os não cadastrados na ESF (35,62%), que avaliam a saúde como regular/ruim/muito ruim (51,38%), que possuem alguma doença crônica (47,51%), não faz uso de bebidas alcoólicas (35,98%), são ex-fumantes (40,31%), consomem verduras menos de cinco vezes por semana (35,48%), de 60 anos ou mais (42,05%), com renda *per capita* acima de três salários mínimos (36,35%), e com obesidade (39,04%). Além disso, a média (em minutos) da prática de atividade física foi de 48,69 (± 1,20) ( Tabela 1 ).

**Tabela 1:** Prevalência de problemas de sono, razão de prevalências bruta e ajustada entre o desfecho e as variáveis sociodemográficas, estilo de vida e condições de saúde da população brasileira. Pesquisa Nacional de Saúde, 2019.

Variáveis	n	Descritiva		Análise bivariada			Análise multivariada		
		Prevalência	IC95%	RP _bruta_	IC95%	p	RP _ajustada_	IC95%	p
Sexo									
Homem	44.752	27,50	26,65–28,36	1			1		
Mulher	49.362	41,84	41,03–42,64	1,52	1,47–1,57	< 0,005	1,41	1,36–1,46	< 0,005
Raça ou cor da pele									
Brancos	34.320	36,15	35,19–37,12	1			1		
Negros	58.390	34,34	33,56–35,11	0,94	0,91–0,98	0,020	0,96	0,92–0,99	0,019
Asiáticos	692	29,42	23,05–36,70	0,81	0,63–1,03	0,100	0,79	0,62–1,01	0,071
Indígenas	702	39,95	32,80–47,56	1,10	0,91–1,33	0,310	1,04	0,88–1,23	0,592
Estado Civil									
Solteiro	42.325	32,04	31,12–32,95	1			1		
Casado	36.354	35,46	34,56–36,36	1,10	1,06–1,14	< 0,005	0,99	0,95–1,02	0,66
Divorciado	7.713	42,81	40,79–44,84	1,33	1,26–1,40	< 0,005	1,05	1,00–1,11	0,027
Viúvo	7.722	46,19	44,31–48,07	1,44	1,37–1,51	< 0,005	0,96	0,91–1,01	0,165
Escolaridade									
Ensino superior	18.287	36,08	34,71–37,46	1			1		
Ensino médio	31.128	31,38	30,38–32,39	0,86	0,83–0,91	< 0,005	0,89	0,85–0,93	< 0,005
Ensino fundamental	36.829	36,94	35,97–37,92	1,02	0,98–1,06	0,270	0,88	0,84–0,92	< 0,005
Sem instrução	7.870	43,52	41,44–45,60	1,20	1,13–1,28	< 0,005	0,94	0,88–1,01	0,120
Aglomeração domiciliar									
1 morador	14.760	41,00	39,72–42,28	1					
2–3 moradores	49.219	36,06	35,31–36,81	0,87	0,84–0,91	< 0,005			
> 4 moradores	30.135	32,97	31,96–33,99	0,80	0,77–0,83	< 0,005			
Local de moradia									
Rural	21.405	30,03	28,95–31,12	1			1		
Urbano	72.709	35,92	35,21–36,63	1,19	1,15–1,24	< 0,005	1,16	1,11–1,20	< 0,005
Cobertura da ESF									
Sim	59.358	35,25	34,52–35,97	1					
Não	23.424	35,62	34,32–36,93	1,01	0,97–1,04	0,589			
Não sabe	11.332	33,03	31,39–34,71	0,93	0,88–0,98	0,015			
Autoavaliação do estado saúde									
Muito boa/boa	60.055	27,65	26,92–28,38	1			1		
Regular/ruim/muito ruim	34.059	51,38	50,34–52,40	1,85	1,80–1,91	< 0,005	1,56	1,51–1,62	< 0,005
Possui DCNT									
Não	37.093	23,15	22,33–23,98	1			1		
Sim	44.125	47,51	46,64–48,37	2,05	1,97–2,13	< 0,005	1,70	1,64–1,78	< 0,005
Uso de álcool									
Não bebe	55.430	35,98	35,24–36,72	1			1		
Uso moderado	20.384	33,92	32,69–35,15	0,94	0,90–0,98	0,003	1,02	0,98–1,07	0,196
Uso excessivo	15.032	33,70	32,17–35,26	0,93	0,89–0,97	0,004	1,14	1,09–1,20	< 0,005
Tabagismo									
Nunca fumou	55.236	32,17	31,44–32,90	1			1		
Ex-fumante	24.224	40,31	39,08–41,54	1,25	1,20–1,29	< 0,005	1,11	1,07–1,15	< 0,005
Fumante	11.386	38,80	37,15–40,47	1,20	1,25–1,26	< 0,005	1,16	1,10–1,22	< 0,005
Consumo de verduras semanal									
≥ 5 vezes	46.754	35,48	34,64–36,33	1			1		
< 5 vezes	44.092	34,64	33,74–35,53	0,96	0,93–0,99	0,029	1,06	1,02–1,09	< 0,005
Idade									
15–29 anos	18.648	27,44	26,18–28,72	1					
30–59 anos	52.322	36,22	35,45–36,99	1,32	1,25–1,38	< 0,005			
≥ 60 anos	23.144	42,05	40,93–43,17	1,53	1,45–1,61	< 0,005			
Renda *per capita*									
Até 1 salário mínimo	10.964	35,18	34,38–35,98	0,96	0,90–1,00	0,186			
De 1 a 3 salários mínimos	31.109	34,60	33,60–35,61	0,95	0,92–1,01	0,062			
Acima de 3 salários mínimos	52.017	36,35	34,70–38,03	1					
IMC									
Desnutrição	2.197	34,47	30,62–38,52	1,05	0,93–1,18	0,366			
Eutrófico	36.356	32,68	31,74–33,62	1					
Sobrepeso	32.972	35,46	34,51–36,42	1,08	1,04–1,12	< 0,005			
Obesidade	18.106	39,04	37,82–40,26	1,19	1,14–1,24	< 0,005			
Atividade física semanal (minutos)	94.114	48,69 ± 1,20	46,33–51,04	1,09	1,03–1,15	0,003			

RP _bruta_: razão de prevalência bruta; RP _ajustada_: razão de prevalência ajustada; IC95%: intervalo de confiança de 95%; ESF: Estratégia Saúde da Família; DCNT: doenças crônicas não transmissíveis; IMC: índice de massa corporal.

A análise da associação entre problemas de sono e as características sociodemográficas, de estilo de vida e saúde dos brasileiros na análise bivariada mostrou que todas as variáveis pesquisadas apresentaram p < 0,20 e foram incluídas no modelo de regressão multivariada ( Tabela 1 ).

No modelo final da análise multivariada, a maior prevalência de problemas de sono foi associado ao sexo feminino (RP = 1,41), aos divorciados (RP = 1,05), aos que vivem no meio urbano (RP = 1,16), aos que avaliam sua saúde como regular⁄ruim⁄muito ruim (RP = 1,56), aos que possuem alguma doença crônica (RP = 1,70), aos que fazem uso excessivo de álcool (RP = 1,14), aos fumantes (RP = 1,16) ou ex-fumantes (RP = 1,11) e com frequência de consumo de verduras menor que cinco vezes por semana (RP = 1,06). Além disso, ser negro (RP = 0,96), ter cursado o ensino médio (RP = 0,89) ou ensino fundamental (RP = 0,88) foram associados à menor prevalência de problemas de sono ( Tabela 1 ).

No que diz respeito ao desfecho ‘uso de medicamentos indutores do sono’, a análise descritiva mostrou que a prevalência desse desfecho foi mais elevada entre o sexo feminino (11,51%), os brancos (10,37%), viúvos (18,32%), entre pessoas sem instrução (13,44%), que moram sozinhos (12,68%), em ambientes urbanos (8,9%), tem cadastro na ESF (8,68%), que avaliam sua saúde como regular/ruim/muito ruim (15,72%), que possui diagnóstico de doença crônica (15,2%), que não usam álcool (10,39%), que são fumantes (10,48%), que consomem verduras cinco vezes ou mais por semana (9,13%), possuem 60 anos ou mais (15,27%), que são obesos (10,54%) e com renda *per capita* acima de três salários mínimos (10,38%). A média (em minutos) da prática de atividade física foi de 53,86 (± 2,47) ( Tabela 2 ).

**Tabela 2:** Prevalência de uso de medicamentos indutores do sono, razão de prevalências bruta e ajustada entre o desfecho e as variáveis sociodemográficas, estilo de vida e condições de saúde da população brasileira. Pesquisa Nacional de Saúde, 2019.

Variáveis	n	Descritiva		Análise bivariada			Análise multivariada		
		Prevalência	IC95%	RP _bruta_	IC95%	p	RP _ajustada_	IC95%	p
Sexo									
Homem	44.752	5,21	4,84–5,59	1			1		
Mulher	49.362	11,51	11,02–12,03	2,21	2,03–2,40	< 0,005	1,57	1,43–1,73	< 0,005
Raça ou cor da pele									
Branco	34.320	10,37	9,79–10,98	1			1		
Negros	58.390	7,18	6,82–7,56	0,69	0,64–0,74	< 0,005	0,72	0,67–0,78	< 0,005
Asiáticos	692	6,10	3,37–10,79	0,58	0,31–1,08	0,088	0,55	0,31–0,98	0,044
Indígenas	702	8,68	5,55–13,33	0,83	0,54–1,29	0,423	0,78	0,53–1,15	0,222
Estado civil									
Solteiro	42.325	5,61	5,21–6,04	1			1		
Casado	36.354	9,06	8,56–9,22	1,61	1,47–1,77	< 0,005	1,03	0,93–1,13	0,536
Divorciado	7.713	15,77	14,31–17,35	2,81	2,48–3,17	< 0,005	1,46	1,30–1,65	< 0,005
Viúvo	7.722	18,32	16,87–19,87	3,26	2,92–3,64	< 0,005	1,19	1,06–1,34	< 0,005
Escolaridade									
Superior	18.287	8,37	7,59–9,22	1			1		
Médio	31.128	6,04	5,57–6,55	0,72	0,63–0,81	< 0,005	0,80	0,71–0,91	0,001
Fundamental	36.829	10,36	9,81–10,93	1,23	1,11–1,37	< 0,005	0,91	0,81–1,02	0,112
Sem instrução	7.870	13,44	12,02–15,01	1,60	1,39–1,85	< 0,005	0,95	0,81–1,12	0,594
Aglomeração domiciliar									
1 morador	14.760	12,68	11,88–13,54	1					
2–3 moradores	49.219	9,68	9,23–10,14	0,76	0,70–0,82	< 0,005			
> 4 moradores	30.135	6,51	6,02–7,04	0,51	0,46–0,57	< 0,005			
Local de moradia									
Rural	21.405	6,37	5,84–6,93	1			1		
Urbano	72.709	8,90	8,54–9,28	1,39	1,27–1,52	< 0,005	1,32	1,21–1,45	< 0,005
Cobertura da ESF									
Sim	59.358	8,68	8,29–9,08	1					
Não	23.424	8,46	7,77–9,20	0,97	0,89–1,06	0,572			
Não sabe	11.332	8,03	7,11–9,07	0,92	0,80–1,06	0,269			
Autoavaliação do estado saúde									
Muito boa/boa	60.055	5,26	4,93–5,62	1			1		
Regular/ruim/muito ruim	34.059	15,72	15,02–16,45	2,98	2,75–3,23	< 0,005	1,79	1,64–1,95	< 0,005
Possui DCNT									
Não	37.093	2,41	2,10–2,77	1			1		
Sim	44.125	15,20	14,59–15,82	6,29	5,42–7,31	< 0,005	4,07	3,48–4,77	< 0,005
Uso de álcool									
Não bebe	55.430	10,39	9,95–10,85	1			1		
Uso moderado	20.384	6,65	6,03–7,33	0,63	0,57–0,71	< 0,005	0,75	0,67–0,84	< 0,005
Uso excessivo	15.032	4,79	4,20–5,45	0,46	0,40–0,52	< 0,005	0,68	0,59–0,78	< 0,005
Tabagismo									
Nunca fumou	55.236	7,41	7,04–7,79	1			1		
Ex–fumante	24.224	10,35	9,69–11,06	1,39	1,28–1,52	< 0,005	1,15	1,05–1,25	0,002
Fumante	11.386	10,48	9,48–11,56	1,41	1,26–1,58	< 0,005	1,49	1,33–1,67	< 0,005
Consumo de verduras por semana									
≥ 5 vezes	46.754	9,31	8,84–9,81	1					
< 5 vezes	44.092	7,64	7,21–8,09	0,81	0,75–0,88	< 0,005			
Idade									
15–29 anos	18.648	3,03	2,58–3,55	1			1		
30–59 anos	52.322	8,70	8,26–9,17	2,87	2,43–3,39	< 0,005	1,50	1,26–1,79	< 0,005
≥ 60 anos	23.144	15,27	14,47–16,10	5,04	4,27–5,96	< 0,005	1,50	1,24–1,81	< 0,005
Renda *per capita*									
Até 1 salário mínimo	10.964	7,94	7,52–8,38	0,76	0,68–0,84	< 0,005			
De 1 a 3 salários mínimos	31.109	8,86	8,29–9,46	0,85	0,76–0,95	0,006			
Acima de 3 salários mínimos	52.017	10,38	9,41–11,44	1					
IMC									
Desnutrição	2.197	7,61	5,91–9,75	1,03	0,78–1,35	0,823			
Eutrófico	36.356	7,38	6,91–7,87	1					
Sobrepeso	32.972	9,05	8,50–9,64	1,22	1,12–1,34	0,006			
Obesidade	18.106	10,54	9,82–11,31	1,42	1,29–1,58	0,006			
Atividade física semanal (minutos)	94.114	53,86 ± 2,47	49,00–58,72	1,18	1,08–1,30	< 0,005			

RP _bruta_: razão de prevalência bruta; RP _ajustada_: razão de prevalência ajustada; IC95%: intervalo de confiança de 95%; ESF: Estratégia Saúde da Família; DCNT: doenças crônicas não transmissíveis; IMC: índice de massa corporal.

A análise da associação entre problemas de sono e as características sociodemográficas, de estilo de vida e saúde dos brasileiros na análise bivariada mostrou que apenas a variável denominada “cobertura da ESF” não apresentou o p < 0,20. Todas as demais variáveis foram incluídas no modelo de regressão multivariada.

No modelo final da análise multivariada, a maior prevalência do uso de medicamentos indutores do sono foi associada ao sexo feminino (RP = 1,57), entre divorciados (RP = 1,46), viúvos (RP = 1,19), que vivem no meio urbano (RP = 1,32), que avaliam sua saúde como regular/ruim/muito ruim (RP = 1,79), com diagnóstico de doença crônica (RP = 4,07), ex-fumante (RP = 1,15), fumante (RP = 1,49), ter 30 a 59 anos (RP = 1,50) e 60 anos ou mais (RP = 1,50). Foram associados à menor prevalência de uso de medicamentos para dormir: ser negro (RP = 0,77), asiático (RP = 0,55), ter o ensino médio (RP = 0,80), que faz uso moderado de álcool (RP = 0,75) ou uso excessivo de álcool (RP = 0,68) ( Tabela 2 ).

## DISCUSSÃO

As prevalências de brasileiros acima de 15 anos que relataram ter problemas no sono, como dificuldade para adormecer, acordar frequentemente à noite ou dormir mais do que de costume, e usaram medicamentos para dormir nas duas semanas anteriores à pesquisa, foram de 35,1% e 8,5%, respectivamente. Essas prevalências são superiores aos encontrados na PNS em 2013, em que 28,9% dos entrevistados relataram apresentar problemas com o sono ^13^ e 7,6% fizeram uso de medicamentos para dormir ^11^ .

Corroborando estudos brasileiros, ambos os desfechos analisados nesta pesquisa foram mais comuns em mulheres ^14^ , em pessoas divorciadas ^15 , 16^ , moradoras na zona urbana ^17^ , que avaliam a sua condição de saúde de forma negativa (regular/ruim/muito ruim) ^16 ,^ , que possuem alguma doença crônica ^16 ,^ e entre fumantes e ex-fumantes.

Os fatores associados às diferenças relacionadas ao sono entre os sexos são bastante discutidos na literatura, contudo, não existe um consenso sobre essa temática ^19^ . Alguns autores relacionam o sono mais fragmentado das mulheres às variações hormonais e fisiológicas ^19^ ou às questões sociais a que as mulheres estão expostas, como maiores cobranças sociais e sobrecarga de trabalho profissional e doméstico ^20^ .

De forma geral, as mulheres relatam maior necessidade de sono e mais queixas subjetivas de sono não restaurador em relação aos homens ^19^ , o que pode ter contribuído para a maior prevalência no uso de medicações. Além disso, as mulheres também apresentam melhor aderência aos tratamentos e maior autocuidado, o que pode ter relação com a maior adesão ao uso de medicamentos ^20^ .

Corroborando outros estudos brasileiros, a maior prevalência de problemas de sono ^15^ e uso de medicamentos indutores do sono ^16^ foi encontrada em pessoas divorciadas. Em revisão de literatura realizado por Lamela ^21^ (2009), o divórcio é considerado um dos estressores de maior impacto na vida adulta, uma vez que as pessoas que passam por esse processo têm mudanças em muitas áreas de suas vidas em um curto período de tempo, tornando-se mais propensas ou vulneráveis a sintomatologias psicológicas. Isso pode explicar a maior prevalência em dificuldade para dormir, acordar frequentemente à noite ou dormir mais do que de costume, bem como uso de medicamentos indutores do sono.

Outro fator encontrado neste estudo que produz impacto no sono dos brasileiros é o fato de morarem em zonas urbanas. A intensidade das luzes artificiais presentes na zona urbana, especialmente no horário noturno, influencia fortemente a duração do sono e os horários de dormir e acordar ^22^ . A presença da luz artificial provoca alterações no organismo, como a supressão da melatonina, hormônio sinalizador da noite e indutor do sono em humanos, e aumento do nível de atividade no sistema nervoso, resultando em maior alerta e consequente dificuldade para adormecer ^17^ .

As maiores prevalências de ambos os desfechos também foram associadas às pessoas que avaliam sua saúde como regular/ruim/muito ruim. A autoavaliação do estado de saúde e a avaliação autorreferida dos problemas de sono estão intimamente relacionadas, ou seja, estar satisfeito com a maneira como se dorme é um dos principais fatores na autoavaliação de saúde ^23^ . Em estudos com jovens brasileiros ^23^ foi observado que acreditar ter um bom sono aumenta a probabilidade de relatar boa autopercepção de saúde.

Em contrapartida, estudo de amostragem representativa da população brasileira com pessoas de 50 anos ou mais observou associação entre a autoavaliação de saúde como ruim com o maior uso de medicações para dormir ^16^ . A mesma pesquisa demonstra que a presença de doenças crônicas esteve associada a maior prevalência de uso de medicamentos ^16^ , o que também foi observado neste estudo.

De forma geral, as doenças crônicas geram inflamações no organismo podendo causar dores, alterações emocionais e outras consequências do não restabelecimento dos sistemas fisiológicos após os eventos de vigília ^24^ . Doenças como hipertensão, osteoporose, artrite/artrose, lombalgia, depressão e obesidade são associadas a problemas de sono ^24^ . Além disso, as pessoas que apresentaram cinco ou mais problemas de saúde concomitantes tiveram uma prevalência 4,19% maior de apresentar problemas de sono, quando comparadas com as pessoas com nenhuma ou menos comorbidades ^18^ .

Os indivíduos que relataram ser fumantes ou ex-fumantes, também apresentaram maior prevalência nos desfechos estudados. A nicotina, principal substância do cigarro, é uma substância estimulante e está associada a modificações na arquitetura do sono, causando a fragmentação e diminuindo a eficiência do sono ^25^ .

Além disso, a maior prevalência de problemas de sono foi associada ao uso excessivo de álcool, o qual tem consequências prejudiciais à saúde geral dos indivíduos. O consumo excessivo de álcool interfere na fisiologia do sono, especialmente na segunda metade da noite, facilitando os despertares, causando um sono fragmentado ^26^ , refletindo em má qualidade do sono.

O resultado de menor prevalência de uso de medicamentos indutores do sono associado a fazer uso moderado ou excessivo de álcool, também foi observado em outro estudo transversal de base populacional brasileira que utilizou a PNS realizada no ano de 2013 ^11^ , seu resultado pode ser decorrente do efeito hipnótico do álcool, que provoca depressão do sistema nervoso central e reduz a latência do sono, facilitando o adormecimento do indivíduo ^26^ .

Outro fator de importante discussão são os hábitos alimentares inadequados e distúrbios do sono, horário de dormir, má qualidade e duração do sono ^27^ . Um exemplo disso, é a associação entre o aumento do consumo de frutas e verduras com a melhoria da qualidade e aumento na duração do sono ^27^ . Portanto, isso pode ter relação com a associação observada neste estudo, de maior prevalência de problemas do sono em indivíduos que relataram frequência de consumo de verduras menor que cinco vezes por semana.

Em relação à idade, foi observado uma associação entre ter mais de 30 anos e maior prevalência do uso de medicamentos indutores do sono. Considerando que a faixa etária de 30 a 59 anos é a que tem maior atividade laboral e social ^2^ , o uso de medicação para dormir pode acontecer na tentativa de uma melhor qualidade de sono, redução de insônia e ansiedade e sensação de relaxamento ^11^ .

Além disso, estudo de base populacional brasileiro realizado com os dados da PNS do ano de 2013 encontrou que 21% dos entrevistados relataram sintomas depressivos e o uso de pílulas para dormir foi associado a pessoas com esses sintomas, o que pode justificar a alta prevalência no uso desses medicamentos ^11^ .

Quanto às pessoas com idade a partir dos 60 anos é observado que fisiologicamente o sono sofre modificações na qualidade, quantidade e arquitetura com o avançar da idade, acarretando um sono mais superficial, fragmentado, com menor duração e de pior qualidade ^28^ .

A terapia farmacológica é a mais utilizada entre o público idoso ^20^ . De forma geral, com uso da medicação, as alterações do sono como a dificuldade de adormecer e os despertares ao longo da noite são minimizados ^16^ . Contudo, muito se discute sobre a utilização em longo prazo dessas medicações e seus efeitos colaterais e sobre a grande quantidade de fármacos consumidos por idosos ^20^ .

Neste estudo, ser negro esteve associado a menor prevalência de problemas de sono e menor prevalência de uso de medicamentos indutores do sono. Esse resultado corrobora estudo epidemiológico realizado nos Estados Unidos em que os adultos brancos relatam tomar mais medicamentos para dormir que os adultos negros ^29^ .

Contudo, a literatura aponta que indivíduos negros têm propensão a pior qualidade do sono, menor duração, maior fragmentação e maior risco de distúrbios respiratórios do sono que indivíduos brancos ^30^ . Apesar desses resultados, pouco se sabe sobre o mecanismo que explica as diferenças étnicas sobre o sono ^30^ .

A menor prevalência de problemas de sono foi também associada a ter cursado o ensino fundamental ou médio, bem como a menor prevalência no uso de medicamentos indutores do sono foi associado a ter cursado o ensino médio. Assim, acredita-se que as pessoas que apresentam maior nível de escolaridade possuam um conhecimento necessário acerca dos comportamentos benéficos à saúde, bem como para melhores hábitos próximos ao horário de dormir, diminuindo as queixas relacionadas ao sono, acarretando menor necessidade de uso de medicações para dormir ^16^ .

Diante do exposto, este estudo apresenta potencialidades importantes, pois trata-se de um estudo nacional representativo de base populacional sobre problemas de sono e uso de medicamentos indutores do sono no Brasil, incluindo mais de 90 mil participantes acima de 15 anos de idade e avaliando condições sociodemográficas, comportamentos do estilo de vida e condições de saúde.

No entanto, uma limitação do estudo foi a impossibilidade de elencar com maior detalhe quais os problemas de sono e quais os medicamentos para dormir utilizados pela população brasileira, tendo em vista que as respostas foram obtidas na PNS de forma autorreferida e sem avaliar as especificações. É importante lembrar que os entrevistadores foram treinados para aplicação dos questionários, porém por serem informações autorreferidas e que questionam comportamentos de semanas anteriores, é possível o viés de memória e diferenças no entendimento dos entrevistados.

Por fim, a prevalência de problemas de sono e o uso de medicamentos indutores do sono foi elevada na população brasileira, sendo um dado importante para a saúde pública, o que constata a necessidade de atenção e cuidado com o sono dessa população. Encontramos nesta análise multivariada, as associações entre as variáveis problemas de sono e uso de medicamentos indutores do sono com diversos fatores sociodemográficos, condições de saúde e estilo de vida. Assim, os resultados deste estudo acrescentam evidências atuais na relação entre o sono e fatores sociais e sugere pesquisas posteriores mais específicas sobre a caracterização dos problemas de sono e tipos, frequência e forma de utilização das medicações para dormir pela população brasileira.
